# Intracapsular Resection of Thoracic Extradural Schwannomas via the Isthmic Approach: Investigation of Clinical Feasibility With 41 Case Series

**DOI:** 10.1111/cns.70506

**Published:** 2025-07-10

**Authors:** Wei Gao, Xinben Hu, Tianjian Liu, Aiqin Chen, Jingyin Chen, Chi Gu, Guangyu Ying, Qiangwei Wang, Yongjian Zhu

**Affiliations:** ^1^ Department of Neurosurgery, The Second Affiliated Hospital Zhejiang University School of Medicine Hangzhou Zhejiang Province China; ^2^ Clinical Research Center for Neurological Diseases of Zhejiang Province Hangzhou China; ^3^ Department of Radiotherapy, The Second Affiliated Hospital Zhejiang University School of Medicine Hangzhou Zhejiang Province China

**Keywords:** dumbbell‐shaped tumor, extradural tumor, schwannoma, surgical technique, thoracic tumor

## Abstract

**Objective:**

Thoracic spinal canal schwannomas can pose surgical challenges when extending into intra‐ and extra‐foraminal regions and the thoracic cavity. This article aims to elucidate the technical nuances and clinical feasibility of the isthmic approach for treating thoracic extradural schwannomas via intracapsular resection.

**Methods:**

The surgical technique was meticulously outlined, and a retrospective analysis of 41 patients who underwent thoracic schwannoma resection via the isthmic approach between January 2014 and August 2022 was conducted. Parameters including gross total resection (GTR) rate, operative duration, estimated blood loss (EBL), incision length, and postoperative hospital stay were evaluated. Preoperative and postoperative neurosurgical functions were assessed using the modified McCormick functional schema and Visual Analogue Scale (VAS).

**Result:**

All patients achieved GTR, with an operative time of 125.37 ± 45.17 min, an average incision length of 6.56 ± 1.04 cm, and an estimated blood loss of 69.88 ± 86.54 mL. The average hospital stay was 6.76 ± 3.73 days. The VAS score significantly decreased postoperatively (preoperative vs. postoperative: 2.10 ± 0.85 vs. 1.32 ± 0.47, *p* < 0.001).

**Conclusion:**

The isthmic approach via intracapsular resection is a promising method for treating extradural schwannomas extending into intra‐ and extra‐foraminal regions. This approach enhances total tumor resection rates, preserves spinal stability, and significantly reduces operative duration, incision length, and blood loss.

## Graphical abstract

A novel minimally invasive surgical approach, the isthmic approach, was introduced for the treatment of thoracic extradural schwannomas. A retrospective analysis of 41 clinical cases demonstrated that this approach improves total tumor resection rates, preserves spinal stability, and significantly reduces operative time, incision length, and intraoperative blood loss.

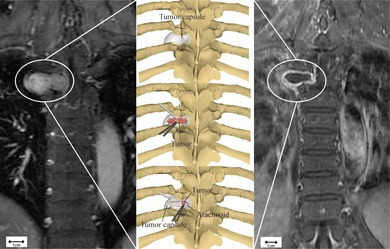

**Abbreviations**
CSF: cerebrospinal fluid
CT: computed tomography
CT‐VRT‐3D reconstruction: computed tomography employing volume rendering technique and 3D reconstruction
EBL: estimated blood loss
GTR: gross total resection
MRI: magnetic resonance imaging
VAS: Visual Analogue Scale

## 1 Introduction

Schwannomas are the most common tumors in spinal canals, and part of them involves both intra and extra foramen during their growth [1, 2, 3]. Eden classified these tumors into four types, called dumbbell‐shaped tumors [4]. Tumors belonging to Eden III and IV types involve extradural, foraminal, and paravertebral regions; when located in thoracic vertebrae, they can make full use of the large space of the chest cavity to expand for a long time without producing clinical symptoms [5, 6]. By the time of detection, these tumors typically attain significant volume, necessitating surgical intervention. Tumors should undergo gross total resection (GTR) without influencing the spinal cord, nerve roots, paraspinal tissues, and organs (including the lungs, thoracic vessels, etc.). There are various approaches available for the removal of such tumors, with strategies often involving either a single posterior approach or a combination with a thoracotomy or thoracoscopic approach. Achieving adequate exposure and ensuring complete tumor resection typically necessitates certain steps in a traditional single posterior surgical approach. These steps may include excision of the facet joint, exposure of the foramen area, and spinal fusion with instrumentation to preserve spinal stability [7, 8, 9, 10]. A combined approach involves repositioning the patient and utilizing dual surgical incisions during the operation, potentially leading to increased iatrogenic trauma [11]. Currently, there is no established optimal method for the removal of those thoracic extradural schwannomas (Eden III and IV types), especially using a single posterior approach without a spinal fusion approach. In this study, we conducted a retrospective review of data from 41 patients diagnosed with thoracic extradural schwannomas who underwent GTR of the tumor utilizing the isthmic approach. The primary objective of this study is to present our institution's single‐center experience with the isthmic approach, outlining technical considerations integral to the surgical procedure, and assessing its clinical feasibility.

## 2 Materials and Methods

### 2.1 Patients

**FIGURE 1:**
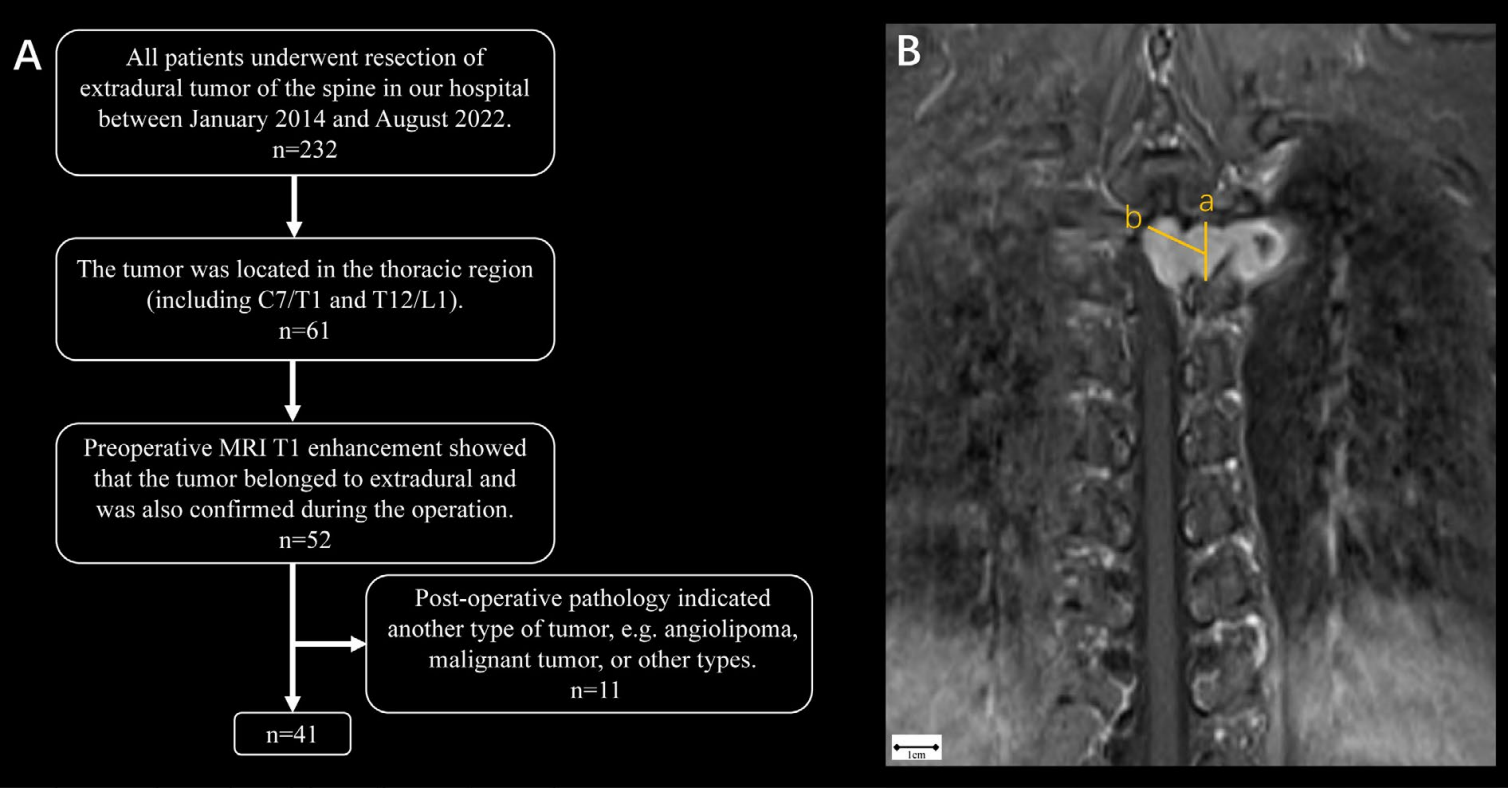
(A) Flow chart depicting patient selection. (B) Criterion for selecting the isthmic approach; The line connecting the centers of the upper and lower facet joints (line a); Distance from the distal end of the intraspinal portion of the tumor to the midpoint of line a (line b).

We retrospectively reviewed 41 patients who underwent thoracic spine extradural schwannoma resection at the second affiliated hospital of Zhejiang University between January 2014 and August 2022 (Figure 1A). Inclusion criteria were: (i) tumor located in the thoracic region (C7/T1 to T12/L1) and diagnosed as schwannoma; (ii) preoperative contrast‐enhanced MRI confirmed an extradural tumor (Eden III or IV), verified during surgery. Exclusion criteria included: (i) postoperative pathology identifying a different tumor type, such as angiolipoma, malignancy, or any other variant. Simultaneously, a criterion for selecting the isthmic approach was established based on our case analysis. When the intraspinal component (line b) exceeded 1.5 cm in length, complete resection via the isthmic approach alone was not feasible, and additional laminectomy was required (Figure 1B). Specifically, a vertical line (line a) was drawn connecting the centers of the upper and lower facet joints of the spinal segment where the tumor was located. The midpoint of line a was then connected to the farthest point of the intraspinal portion of the tumor, forming line b. The length of line b was measured, and a diameter of less than 1.5 cm was considered an indication for proceeding with this surgical approach.

### 2.2 Operative Techniques

#### 2.2.1 Position and Anesthesia

Under general anesthesia, the patients were intubated with an endotracheal tube and positioned in the prone position.

#### 2.2.2 Surgical Techniques

**FIGURE 2:**
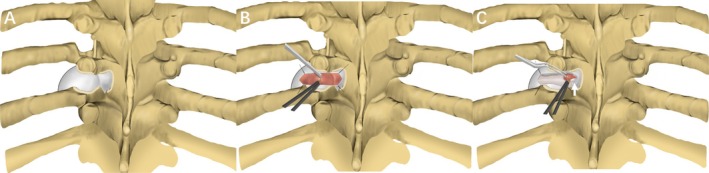
Surgical diagrams illustrating the isthmic approach operation process. (A) The lateral margin of the transverse process was exposed through the intermuscular space between the longissimus muscles and the multifidus. (B) Following suspension of both sides of the tumor capsule, the tumor located in paravertebral is resected entirely through intracapsular resection. (C) Residual tumor within the enlarged intervertebral foramen and intraspinal region was gently delivered and resected along its boundary. Focal thickening of the arachnoid membrane was observed at the tumor–arachnoid interface after resection (white arrow).

For patients with extradural schwannomas classified as Eden III types, following confirmation of the surgical segment via C‐arm (Philips, BV Pulsera) fluoroscopic guidance, a longitudinal incision was marked over the tumor projection on the skin, situated 3 cm away from the posterior median line and parallel to the spine. The length of the paravertebral incision was determined to be sufficient for adequate exposure of the tumor and the intervertebral foramen. Following incision of the skin and latissimus dorsi muscle, the lateral margin of the transverse process was exposed through the intermuscular space between the longissimus muscles and the multifidus using a retractor. Depending on the tumor's location and size, a portion of the transverse process, ribs, or isthmus was removed using a Midas‐Rex drill (Medtronic) (Figure 2A). Once fully exposed, the tumor surface was cauterized using a Bipolar Electrocoagulator under the microscope. The tissue surrounding the tumor formed a capsule on its surface. Consequently, the outermost tumor capsule was incised along the direction of the nerve root and delicately separated from the tumor using a nerve stripper. Suspension of both sides of the tumor capsule provided gentle traction, facilitating a clear surgical field for the surgeon. Subsequently, intratumoral decompression was initiated through partial resection of the tumor, followed by separation of the residual tumor along with the inner layer of the capsule using a nerve stripper for piecemeal resection (Figure 2B). When the paravertebral tumor was completely excised, the operation microscope was adjusted for better visualization of the intervertebral foramen area. Depending on the size of the intraspinal tumor—particularly in cases where part of the tumor is obscured by the pedicle (as shown in Figure S1A,C)—the isthmic space can be further enlarged using a grinding drill (Figure S1F). Additionally, sutures were used to suspend the tumor capsule within the intervertebral foramen area. Subsequently, any remaining tumor within the enlarged intervertebral foramen and intraspinal region was gently delivered and resected along its boundary. After tumor resection, focal thickening of the arachnoid membrane was noted at the tumor–arachnoid interface (Figure 2C, Figure S2A,B, Video S1). Finally, the tumor cavity was filled with a gelatin sponge, the muscle layers were closed with absorbable sutures, and the skin was closed with intradermal sutures. For patients with tumors classified as Eden IV types, complete tumor resection can typically be achieved without the need for bone destruction at the foraminal area. If CSF leakage is identified or suspected during tumor resection, we first repair any dural defects using autologous fat, fascia, or artificial dura as appropriate. Following this, a watertight suture of the tumor capsule is performed to further prevent postoperative CSF leakage. Additionally, in patients with pleural disruption during tumor dissection, the pleural defect should be meticulously sutured under microscopic guidance. A drainage tube should also be placed within the tumor cavity to reduce the risk of pleural effusion and fluid accumulation in the surgical area.

#### 2.2.3 Postoperative Measurements

In some patients, a drainage tube was utilized, with the majority being removed within 2 days postoperatively. Most patients were discharged within 4–8 days following surgery, unless lumbar puncture results suggested the presence of intracranial infection, necessitating further antibiotic treatment until the disease was controlled before discharge.

### 2.3 Outcome Measurements and Data Collection

Neurosurgical condition and pain were assessed with the modified McCormick functional schema and Visual Analogue Scale (VAS) before surgery and at discharge. Routine postoperative contrast‐enhanced MRI assessed tumor resection. At 6 months, contrast‐enhanced MRI and CT with Volume Rendering Technique (VRT) and 3D reconstruction evaluated tumor recurrence and bone changes. Follow‐ups included annual contrast‐enhanced MRI and CT‐VRT‐3D assessments. Outcome data include tumor classification, size, location, surgical timing, estimated blood loss (EBL), incision length, hospital stay, follow‐up duration, McCormick and VAS scores, and spinal stability.

### 2.4 Statistical Analysis

All data were analyzed by SPSS 23.0 (IBM, Armonk, NY, USA). Continuous variables were presented as mean values ± standard deviations (SD). In the one‐way analysis, the Shapiro–Wilk test was first conducted to evaluate the normality of data within each group, while the *F*‐test was used to assess the homogeneity of variances. When the continuous variables satisfied both the normality and variance homogeneity assumptions, a *t*‐test was applied to compare differences between groups. If either assumption was violated, the Mann–Whitney *U* test was used as an alternative. The Wilcoxon signed‐rank test was used to compare the preoperative and postoperative scores (McCormick and VAS) due to the non‐normal distribution of the paired data. *p* < 0.05 was considered statistically significant.

## 3 Results

### 3.1 Patient Characters

The clinical data of the 41 patients are summarized in Table 1. Of these patients, 26 were male and 15 were female, with an average age of 48.56 ± 12.58 years. Tumor distribution among the patients revealed that 7 tumors were located in the upper thoracic segment (above T4), 11 were found in the middle thoracic region (T5‐T8), and 23 were situated in the lower thoracic region and thoracolumbar junction (T9‐L1). The average tumor size was 37.68 ± 10.07 mm, with one tumor exhibiting a maximum diameter in the coronal position exceeding 70 mm. Based on Eden's classification, 20 cases were categorized as type III, while 21 were classified as type IV.

**TABLE 1:** Demographic and clinical characteristics of patients.

	Baseline	Postoperative	*p*
Gender			
Male	26		
Female	15		
Age (years old)	48.56 ± 12.58		
Eden's classification			
Type III	20		
Type IV	21		
Tumor size (maximum diameter in coronal position, mm)	37.68 ± 10.07		
Tumor site			
C7‐T4	7		
T5‐T8	11		
T9‐L1	23		
Operation result			
Time of operation (min)	125.37 ± 45.17		
Estimated blood loss (mL)	69.88 ± 86.54		
Length of incision (cm)	6.56 ± 1.04		
Postoperative hospital stay (day)	6.76 ± 3.73		
Follow‐up (month)	21.98 ± 10.91		
McCormick score	1.17 ± 0.38	1.05 ± 0.22	0.06^a^
VAS score	2.10 ± 0.85	1.32 ± 0.47	< 0.001***^,^ ^a^

*Note:* Data are expressed as Mean ± SD. Statistical significance is denoted as ***p < 0.001.

Abbreviation: VAS, Visual Analogue Scale.

^a^ Groups were compared using the Wilcoxon signed‐rank test.

### 3.2 Surgical and Followed‐Up Data

The operation duration averaged 125.37 ± 45.17 min, with an average incision length of 6.56 ± 1.04 cm. Estimated blood loss during surgery was 69.88 ± 86.54 mL, and the average postoperative hospital stay was 6.76 ± 3.73 days (Table 1). Postoperative MRI examinations confirmed that all patients achieved GTR. Pathological analysis revealed benign schwannomas in all cases. The VAS score exhibited a significant decrease postoperatively compared to preoperative levels (preoperative vs. before discharge: 2.10 ± 0.85 vs. 1.32 ± 0.47, *p* < 0.001). The mean follow‐up duration for all patients was 21.98 ± 10.91 months, during which no instances of tumor recurrence or spinal instability were observed.

### 3.3 Complications

No occurrences of lung injury, nor significant impairment of neurosurgical function, were reported in patients following the operation. When parietal pleura injury was suspected intraoperatively, a drainage tube was placed to evacuate fluid from the surgical cavity and reduce the risk of postoperative effusion. However, three out of the 41 patients developed pleural effusion post‐surgery. Among them, two had mild effusion volumes ranging from 100 to 200 mL, which were self‐limiting and resolved with conservative treatment. In the remaining case, ultrasound evaluation estimated the effusion volume to exceed 600 mL. After consultation with the thoracic surgery team, a closed thoracic drainage tube was placed, leading to clinical improvement. Additionally, one patient developed an infection at the incisional wound postoperatively. The patient successfully recovered and was discharged from the hospital after receiving antibiotic treatment.

### 3.4 Illustrative Case

#### 3.4.1 Case I

**FIGURE 3:**
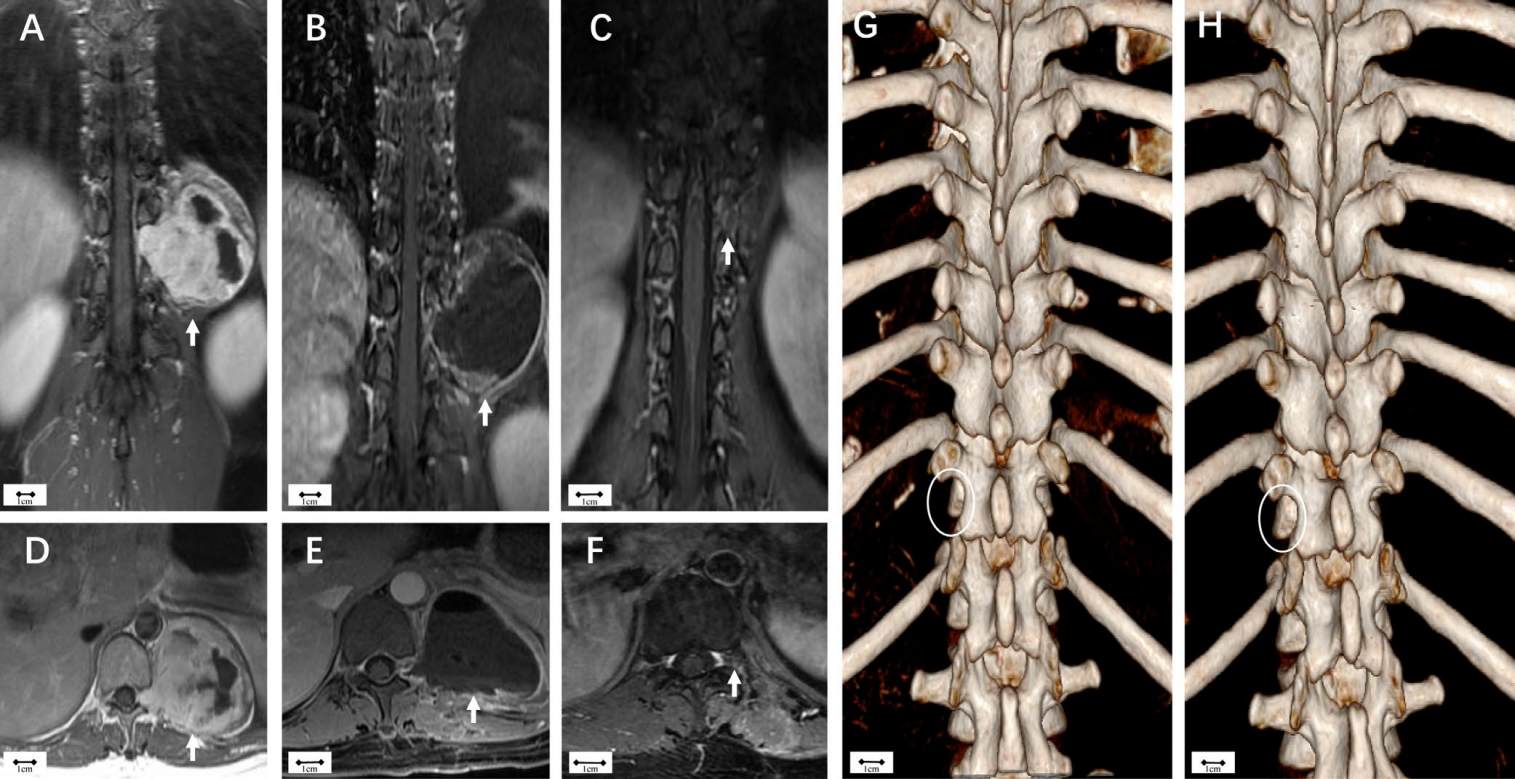
Case I (Eden type IV). (A, D) Preoperative enhanced MRI showed the tumor on the left side of T11/12. (B, E) Postoperative enhanced MRI showed that the tumor was resected entirely through intracapsular. (C, F) The tumor capsule was absorbed, and no recurrence occurred during the 2‐year follow‐up period. (G, H) Postoperative and 2‐year follow‐up CT‐VRT‐3D reconstructions revealing no impact on the spinal bone integrity (circle).

A 39‐year‐old woman was incidentally found to have a paravertebral mass during a health check‐up. Neurological examination was normal. CT showed a smooth‐edged lesion with left T11/12 foraminal enlargement. MRI revealed a tumor with iso‐intense signal on T1‐weighted images and uneven enhancement on contrast MRI (Figure 3A,D). An extradural schwannoma (Eden IV) was provisionally diagnosed. The tumor was resected via the isthmic approach. Through the intermuscular plane, the left T11/12 transverse process was exposed, revealing the tumor ventral to the ribs. The tumor capsule was incised, and the paravertebral component was excised piecemeal, followed by removal of the foraminal component. Postoperative pathology confirmed schwannoma. Postoperative MRI showed no residual tumor (Figure 3B,E), and a 2‐year follow‐up MRI confirmed no recurrence (Figure 3C,F). The operation preserved spinal bone integrity (Figure 3G,H).

#### 3.4.2 Case II

**FIGURE 4:**
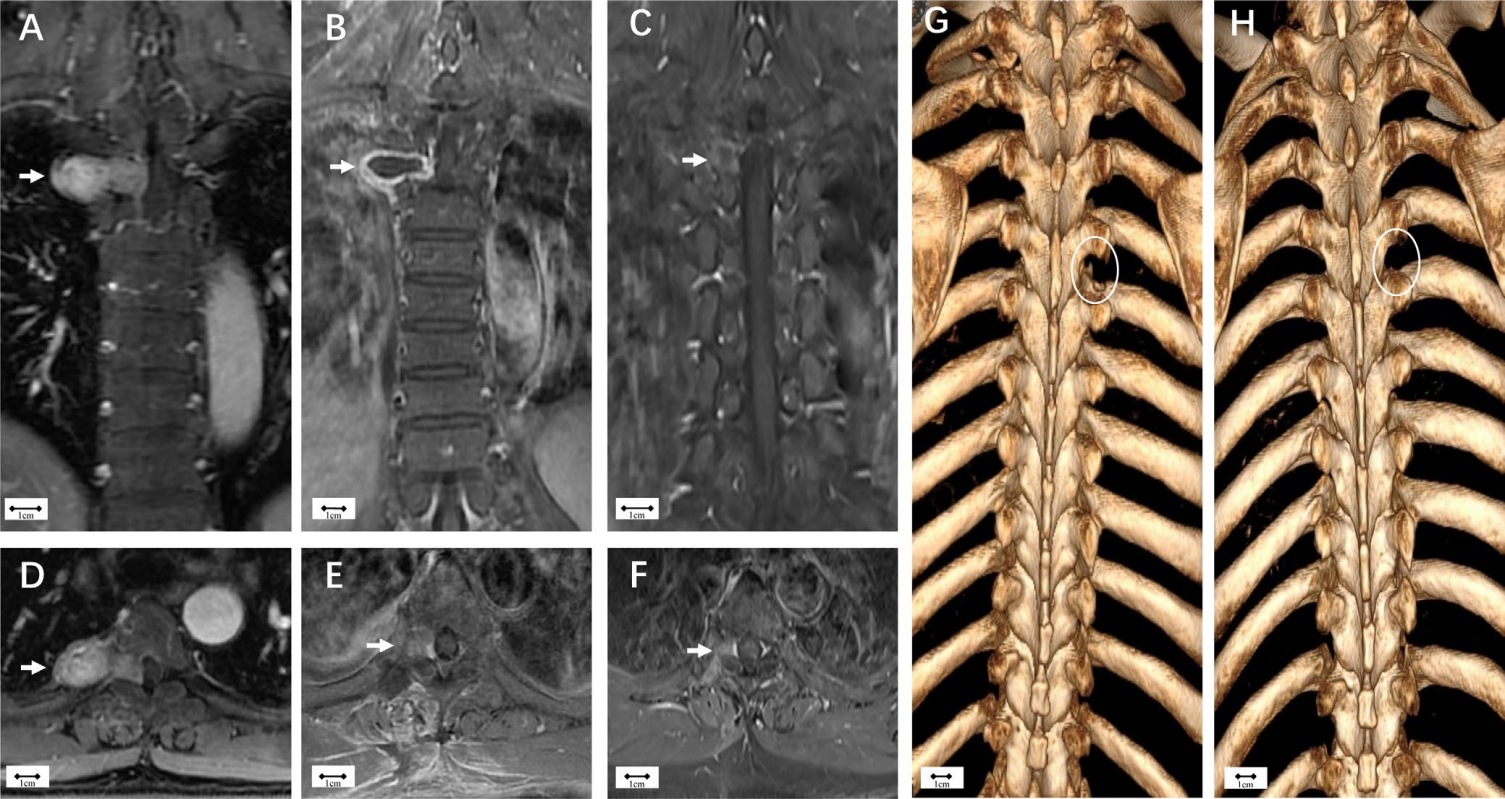
Case II (Eden type III). (A, D) Preoperative enhanced MRI showed a 40 mm × 21 mm × 18 mm mass near the T4/5 spinal column with uneven enhancement. (B, E) Postoperative enhanced MRI showed no tumor residue. (C, F) There was no tumor recurrence at 1‐year follow‐up. (G) Postoperative CT‐VRT‐3D reconstruction indicating destruction of a few ribs, isthmus, and transverse processes while the articular processes remained intact (circle). (H) Follow‐up 1 year later showed partial regeneration of local bones (circle).

A 56‐year‐old male patient presented with right‐sided chest and back pain persisting for 2 years. Neurological examination upon admission revealed no abnormalities. MRI revealed a mass measuring 40 mm × 21 mm × 18 mm near the T4/5 spinal column with uneven enhancement. Coronal MRI demonstrated compression of the thoracic spinal cord on the right side (Figure 4A,D). The tumor was resected via the isthmic approach. During the operation, the lateral edge of the T4/5 transverse process was exposed. Given the tumor involvement of the epidural space, the intervertebral foramen was enlarged using high‐speed drilling before complete tumor resection along the tumor capsule. Postoperative contrast‐enhanced MRI showed no residual tumor (Figure 4B,E), and no recurrence was observed at the 1‐year follow‐up (Figure 4C,F). Postoperative CT‐VRT‐3D reconstruction indicated destruction of a few ribs, isthmus, and transverse processes, while the articular processes remained intact (Figure 4G). Follow‐up 1 year later revealed partial regeneration of local bone structures (Figure 4H).

## 4 Discussion

Extradural schwannoma in the thoracic spine has particular anatomical structures; the paravertebral part of the schwannoma can protrude into the thoracic cavity for growth, which is not possessed by other parts of the schwannoma [6, 12]. It can grow wantonly with the help of the vast space of the chest, resulting in the relatively large volume of the tumor when it was found (4 of the 41 patients included in this study, the maximum diameter in the coronal position of the tumor is more than 50 mm), which poses a significant challenge to the choice of surgical approach and GTR. Only a few studies have previously reported outcomes of this kind of extradural schwannoma in the thoracic region (Table 2). In the early stages, some scholars utilized laminectomy combined with thoracotomy for tumor removal; however, the extensive surgical trauma associated with this combined approach limited its widespread adoption [17, 18]. Subsequently, posterior laminectomy combined with video‐assisted thoracoscopic surgery (VATS) emerged as a viable option for the resection of thoracic extradural schwannomas, particularly those with large volumes extending into the chest cavity. While cases treated with this combined surgical approach achieved favorable clinical outcomes, comparative analysis revealed that, in contrast to the posterior approach, the combined approach did not demonstrate significant advantages in terms of operation time and estimated blood loss [5, 11, 15, 16, 19, 20]. Nam et al. [11] reported Anhydrosis in one case and atelectasis in the other after combined surgery. Also, changing body position during combined surgery may increase the risk of accidental extubation and the time of general anesthesia, especially for elderly patients with poor primary conditions [12, 21]. In addition, the damage to the stellate ganglia caused by transthoracic surgery may lead to Horner's syndrome [5]. On the other hand, some scholars have attempted to remove extradural schwannoma through posterior surgery [9, 22, 23]. Traditional methods involved destroying bony structures such as the lamina, articular surface, and ribs to achieve improved tumor exposure. Subsequently, instrumented fusion was often performed to enhance spinal stability. However, these approaches were associated with increased surgical trauma, bleeding, and extended hospitalization durations, consequently leading to elevated hospitalization costs [24]. Recently, minimally invasive surgical approaches have been widely used to remove spinal schwannomas. Nzokou et al. [13] and Zairi et al. [14] tried to use a tubular retractor for extradural resection in the thoracic spine, but both of them left a small part of the tumor in each case.

**TABLE 2:** Comparison of outcomes reported in previous studies and the present study.

	Number (III, IV)/total	Operation time (min)	Incision length (cm)	Estimated blood loss (ml)	Length of stay (days)	Facetectomy	Instrumented fusion
Posterior approach							
Ando et al. (2013) [9]	5/16	278.60 ± 91.28	NR	555.00 ± 278.54	NR	Yes	Yes
Nzokou et al. (2013) [13]	9/13	193.00 ± 196.74^b^	2.00 ± 1.26	180.00 ± 189.08^b^	2.00 ± 1.42^b^	No	No
Li et al. (2016) [8]	2	160.00 ± 14.14	3.10 ± 3.14	225.00 ± 35.36	4.50 ± 2.12	Yes	Yes
Zairi et al. (2017) [14]	5	219.00 ± 183.21	2.50	230.00 ± 178.89	NR	No	No
Li et al. (2018) [15]	7/12	222.10 ± 4.60^d^	NR	400.80 ± 224.80^d^	8.00 ± 2.40^d^	Yes	Yes
Rong et al. (2018) [10]	12/14	280.00 ± 41.78	5.68 ± 0.59	562.50 ± 459.31	NR	Yes	No
Combined approach							
Nam et al. (2017) [11]	7	344.29 ± 59.54	NR	348.57 ± 277.33	NR	No	No
Li et al. (2018) [15]	13/20	305.30 ± 76.40^c^	NR	618.80 ± 215.6^c^	11.00 ± 1.90^c^	Yes	Yes
Chen et al. (2019) [5]	6/20	244.30 ± 92.00^a^	NR	359.50 ± 211.90^a^	4.10 ± 1.90^a^	NR	No
Harrison et al. (2021) [16]	7	170.71 ± 63.47	NR	142.86 ± 255.78	5.43 ± 3.64	NR	No
Isthmic approach							
This study	41	125.37 ± 45.17	6.56 ± 1.04	69.88 ± 86.54	6.56 ± 1.04	No	No

*Note:* Data are expressed as Mean ± SD.

Abbreviation: NR, value not reported.

^a^ Value calculated with 10 additional cases, not laminectomy and video‐assisted thoracoscopic surgery (VATS) (isolated VATS, thoracotomy plus laminectomy, supraclavicular approach, and supraclavicular approach plus thoracotomy), and four type II cases.

^b^ Value calculated with four additional cases, which were type II.

^c^ Value calculated with seven additional cases, which were type II.

^d^ Value calculated with five additional cases, which were type II.

In this study, we employed an isthmic approach for resecting extradural schwannomas of the thoracic spine. The selection of the surgical approach was contingent upon the anatomical location of the schwannoma. Hence, meticulous preoperative imaging analysis played a pivotal role in identifying the precise tumor location and guiding the choice of surgical approach. Xin et al. [25] analyzed the dura mater and arachnoid structure surrounding nerve roots in seven adult cadavers, as well as the peritumoral structure during surgery. They proposed that for intra‐ and extraforaminal schwannomas involving the subarachnoid space, three stenoses within the tumor were identified: the limitation of the arachnoid sleeve, the limitation of the dural sleeve, and the limitation of the intervertebral foramen. Therefore, preoperative contrast‐enhanced MRI in both coronal and axial planes may aid in preliminarily assessing the relationship between the tumor, dura mater, and subarachnoid space, although definitive confirmation still relies on intraoperative exploration. In addition, the diameter of tumors extending into the intraspinal region was assessed. For tumors where the diameter of line b exceeded 1.5 mm (Figure 1B), a hemi‐laminotomy was performed to excise the intraspinal portion. This approach was chosen due to the limited space within the intervertebral foramen, allowing preservation of the upper and lower facet joints, which are crucial for maintaining spinal stability. During surgery, the intraspinal tumor was bluntly separated along the thickened arachnoid boundary, with the epidural arachnoid bulge serving as a marker for complete resection of the intraspinal tumor.

Schwannoma, being a benign tumor, typically exhibits reduced recurrence rates following GTR. Most intraspinal schwannomas originate from the dorsal sensory nerve roots of the spinal cord, occupying various spaces including the subarachnoid, extradural, foraminal, and paravertebral regions based on their origins and growth direction [14, 19]. In cases involving the thoracic vertebra, a portion of the tumor may extend into the chest cavity, exerting compression on lung tissue and adjacent organs. The pleura enveloping the tumor protruding into the chest cavity forms a tumor capsule with surrounding tissue, facilitating the use of a nerve dissector for tumor dissection [26]. Consequently, intracapsular resection of the tumor can minimize damage to the pleura and achieve GTR. Conversely, complete removal of the tumor capsule may result in chest wall defects and increase the risk of CSF leakage and pleural effusion. Furthermore, Stone et al. [27] conducted a retrospective analysis of 36 patients with schwannoma who underwent pathological examination of the tumor capsule following resection. After an average follow‐up period of 3.1 years, no instances of tumor recurrence were detected through clinical and imaging evaluations. Histopathological examination revealed that the capsule primarily comprised dense, low‐cell collagen, occasionally containing arteries, veins, and nerve bundles. Similarly, in our study, pathological examination of the tumor capsule confirmed the absence of tumor components. Hence, meticulous differentiation of membranous structures and intracapsular tumor resection are crucial for minimizing residual tumor and safeguarding adjacent vital organs and tissues. In addition, the intact arachnoid membrane served as a natural barrier between the tumor and the subarachnoid space, further reducing the risk of tumor dissemination.

There exists considerable debate regarding the requirement for fixation and reconstruction of the thoracic spine following the removal of extradural schwannomas. Ozawa et al. [28] reported that approximately 55% of dumbbell‐shaped tumors necessitate removal from the articular surface during surgical intervention. In such cases, ensuring spinal stability poses a significant challenge, particularly when multi‐stage laminectomy or total facet joint resection is performed. Some scholars believe instrumented fusion should be carried out simultaneously in radical dumbbell‐shaped tumor resection [15]. Among the 41 patients in this study, no thoracic instability or deformity happened during the follow‐up time (8–51 months). The minimal impact on thoracic spine stability can be attributed to several factors. Firstly, extradural tumors of the thoracic spine typically grow laterally, primarily along the intervertebral foramen, and schwannomas rarely cause bone destruction. Secondly, during surgery, we accessed the tumor through the longissimus and multifidus muscle space, resulting in less damage to the paraspinal muscles and ligaments. Most importantly, the isthmic approach necessitates minimal bone removal or no destruction of bony structures to achieve tumor resection, making it undoubtedly the most effective method for preserving stability [29, 30]. Moreover, for patients who undergo hemi‐laminectomy due to either a large intraspinal tumor or intraoperative confirmation of communication between the tumor and the subarachnoid space, we recommend close postoperative follow‐up to monitor spinal stability. If spinal instability is identified during follow‐up, timely secondary fixation should be considered after careful evaluation.

This study analyzed the clinical feasibility of the isthmic approach in treating extradural schwannomas classified as Eden types III and IV. For Eden IV tumors, complete resection can be achieved without disrupting the spinal bone structure by strictly adhering to the intracapsular resection strategy. Similarly, for Eden III tumors that extend into the spinal canal, total resection can be accomplished by enlarging the isthmic space. Several limitations must be noted. First, the isthmic approach is inappropriate for Eden types I and II tumors, which require laminectomy for effective intradural tumor resection. Second, the approach's feasibility for tumors located more than 1.5 cm from the medial foramen remains uncertain and needs further investigation. Lastly, the study's single‐center nature may limit generalizability, and its retrospective design could introduce sample selection bias, impacting the robustness of the findings.

## 5 Conclusion

There exist numerous surgical approaches for resecting schwannomas located in the lateral intervertebral foramen of the thoracic spine, each with its own limitations. However, the isthmic approach, employing intracapsular resection, offers several advantages. This modified approach allows for a one‐stage, single‐incision operation, avoiding the destruction of the articular process and obviating the need for instrumented fusion. Given these benefits, the isthmic approach represents a promising surgical technique deserving of further study and wider adoption.

## Disclosure

The authors have nothing to report.

## Ethics Statement

This study was performed in line with the principles of the Declaration of Helsinki. Approval was granted by the Ethics Committee of the second affiliated hospital of Zhejiang University (2020‐429).

## Conflicts of Interest

The authors declare no conflicts of interest.

## Supporting information


**Figure S1.** Typical tumor partly hidden by the pedicle. (A, C) Preoperative contrast‐enhanced MRI revealed an intra‐ and extra‐foraminal tumor (white arrow) located at the left T10–11 segment. (B, D) Postoperative contrast‐enhanced MRI following resection via the isthmic approach demonstrated gross‐total tumor removal (white arrow). (E–G) CT‐VRT‐3D reconstruction showed that only partial drilling of the transverse process and isthmic bone was performed during surgery (circle‐surgical area; white arrow‐facet joint and pedicle).
**Figure S2.** (A) After the intraspinal portion of the tumor was delivered, a clear boundary between the tumor and the arachnoid membrane was visible, allowing for blunt dissection and subsequent tumor resection; (B) A thickened arachnoid membrane was observed at the site of tumor attachment after resection.

Video S1.

## Data Availability

The datasets generated during and/or analyzed during the current study are available from the corresponding author on reasonable request.
