# Supplementary material for: Intracapsular Resection of Thoracic Extradural Schwannomas via the Isthmic Approach: Investigation of Clinical Feasibility With 41 Case Series
Source: CNS Neurosci Ther. 2025 Jul 10;31(7):e70506. doi: 10.1111/cns.70506 (PMC12241823; doi:10.1111/cns.70506)
Supplement: Supplementary file 1 — Figure S1. Typical tumor partly hidden by the pedicle. (A, C) Preoperative contrast‐enhanced MRI revealed an intra‐ and extra‐foraminal tumor (white arrow) located at the left T10–11 segment. (B, D) Postoperative contrast‐enhanced MRI following resection via the isthmic approach demonstrated gross‐total tumor removal (white arrow). (E–G) CT‐VRT‐3D reconstruction showed that only partial drilling of the transverse process and isthmic bone was performed during surgery (circle‐surgical area; white arrow‐facet joint and pedicle). Figure S2. (A) After the intraspinal portion of the tumor was delivered, a clear boundary between the tumor and the arachnoid membrane was visible, allowing for blunt dissection and subsequent tumor resection; (B) A thickened arachnoid membrane was observed at the site of tumor attachment after resection. [file CNS-31-e70506-s001.docx]

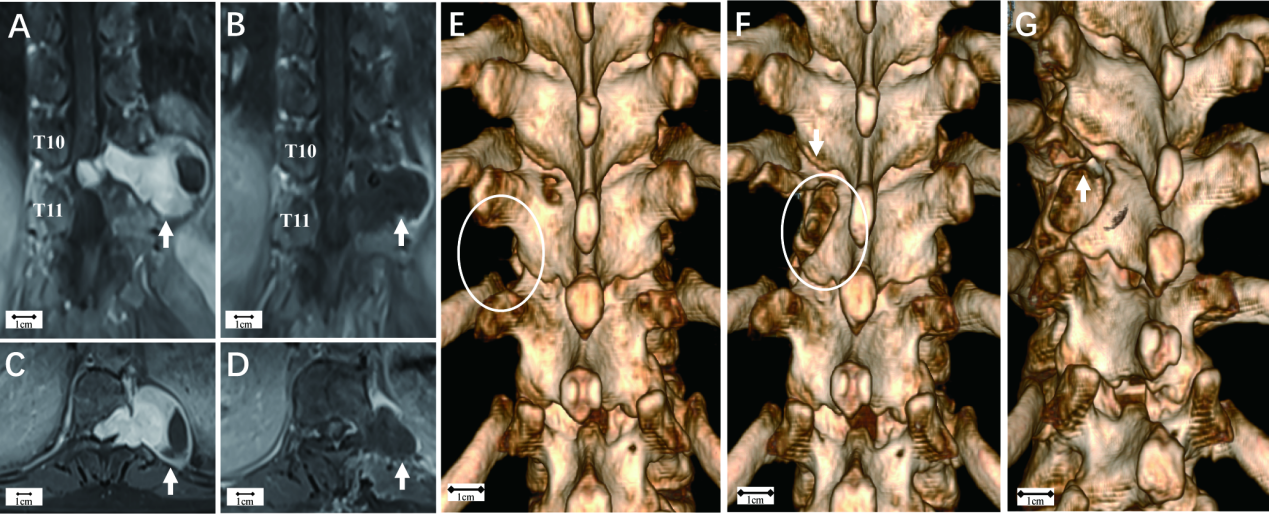


Supplementary Figure 1. Typical tumor partly hidden by the pedicle. AC. Preoperative contrast-enhanced MRI revealed an intra- and extra-foraminal tumor(white arrow) located at the left T10–11 segment. BD. Postoperative contrast-enhanced MRI following resection via the isthmic approach demonstrated gross-total tumor removal(white arrow). EFG. CT-VRT-3D reconstruction showed that only partial drilling of the transverse process and isthmic bone was performed during surgery(circle-surgical area;white arrow-facet joint and pedicle).

MRI, Magnetic Resonance Imaging;

CT-VRT-3D reconstruction, Three‑dimensional reconstruction using computed tomography–volume rendering technique.


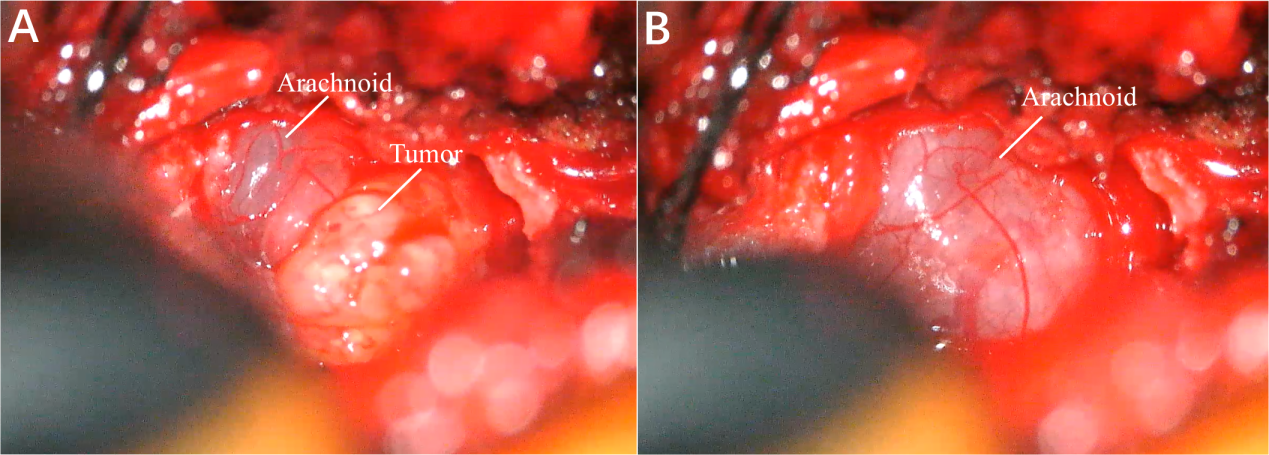
Supplementary Figure 2. A. After the intraspinal portion of the tumor was delivered, a clear boundary between the tumor and the arachnoid membrane was visible, allowing for blunt dissection and subsequent tumor resection; B. A thickened arachnoid membrane was observed at the site of tumor attachment after resection.
